# The effectiveness of the modified Epley maneuver for the treatment of posterior semicircular canal benign paroxysmal positional vertigo

**DOI:** 10.3389/fneur.2023.1328896

**Published:** 2023-12-21

**Authors:** Xiaosu Chen, Jiesheng Mao, Hua Ye, Luping Fan, Qiaowen Tong, Hehui Zhang, Chengcheng Wu, Xiaokai Yang

**Affiliations:** ^1^Neurology Department, Third Affiliated Hospital of Shanghai University, Wenzhou Third Clinical Institute Affiliated to Wenzhou Medical University, Wenzhou People’s Hospital, Wenzhou, China; ^2^Rehabilitation Department, Third Affiliated Hospital of Shanghai University, Wenzhou Third Clinical Institute Affiliated to Wenzhou Medical University, Wenzhou People’s Hospital, Wenzhou, China

**Keywords:** BPPV, otoconia, Epley maneuver, nystagmus, simulation

## Abstract

**Objective:**

To compare the repositioning effect of the modified Epley maneuver and the traditional Epley maneuver for posterior semicircular canal benign paroxysmal positional vertigo (PC-BPPV).

**Methods:**

Sixty-five patients with unilateral PC-BPPV were randomly divided into two groups. The control group received the traditional Epley maneuver, while the experimental group received the modified Epley maneuver, which prolonged the time in the healthy side lying position and the final bowing position. The number of successful repositions after one, two, and three attempts and the total number of successful repositions were recorded and compared between the two groups. A BPPV virtual simulation model was used to analyze the mechanism of the modified Epley maneuver.

**Results:**

The first repositioning success rate of the experimental group was significantly higher than that of the control group (85% vs. 63%, *p* = 0.040). The experimental group achieved 100% repositioning success rate after two attempts, while the control group needed three attempts to reach 86% repositioning success rate. Four cases in the control group experienced canal switching during the repositioning process, while none in the experimental group did. The BPPV virtual simulation model showed that the modified Epley maneuver could facilitate the passage of otoliths through the posterior arm of the posterior semicircular canal, especially through the location of obstruction.

**Conclusion:**

The modified Epley maneuver is more effective than the traditional Epley maneuver in improving the single repositioning success rate and reducing the canal switching rate for PC-BPPV. This study provides a new option for the treatment of BPPV.

## 1 Introduction

Benign paroxysmal positional vertigo (BPPV) stands out as a prevalent cause of peripheral vertigo, constituting 17–42% of reported cases (1). Manifesting as brief episodes of vertigo and nystagmus, BPPV is triggered by alterations in head position relative to gravity, such as lying down, turning over, or standing up (2). The prevailing pathophysiological understanding attributes BPPV to the detachment of otoconia from the utricular macula, migrating into one or more semicircular canals. This migration disrupts normal endolymph flow and induces abnormal stimulation of the cupula (3). BPPV is further categorized based on the involved semicircular canal, with posterior canal BPPV (PC-BPPV) being the most prevalent, accounting for 80% of cases (4).

Diagnosis relies predominantly on patient history and positional tests, such as the Dix-Hallpike test for PC-BPPV and the supine roll test for horizontal canal BPPV (HC-BPPV) (5). Treatment primarily revolves around repositioning maneuvers, aiming to relocate otoconia from the affected semicircular canal back to the utricle through a series of head movements (6). The widely adopted Epley maneuver, introduced by John Epley in 1992 (6, 7), has demonstrated efficacy and safety for PC-BPPV, with success rates ranging from 63.65 to 98% after one or more attempts (8).

Despite its success, some patients exhibit poor response or canal switching, converting PC-BPPV to HC-BPPV during or after the maneuver (9). Factors contributing to these challenges remain not fully elucidated, potentially involving anatomical variations, membranous canal stenosis, otolith adhesion, otolith re-entry, incorrect diagnosis, or inadequate repositioning techniques (10). Consequently, modifications to the Epley maneuver have been proposed to enhance efficacy and reduce adverse effects, including head shaking, prolonged postural holding, or hastened head movements (11). However, these modifications may introduce limitations such as increased complexity, discomfort, or an elevated risk of canal switching (12).

This study introduces a novel modification to the Epley maneuver for PC-BPPV, incorporating a BPPV virtual simulation model. Our modification involves extending the retention time in the healthy lateral position and the final low head position, facilitating the passage of otoliths through the posterior arm of the posterior semicircular canals, especially through obstructed regions. We hypothesize that our modified Epley maneuver can enhance the single repositioning success rate for PC-BPPV compared to the traditional Epley maneuver. To test this hypothesis, we conducted a randomized controlled trial involving 65 patients with unilateral PC-BPPV, comparing repositioning outcomes between the modified and control groups. Additionally, we utilized a BPPV virtual simulation model to analyze the mechanism underlying our modified Epley maneuver. The aim of this study is to provide a promising treatment option for PC-BPPV, especially for refractory PC-BPPV.

## 2 Materials and methods

### 2.1 Sample size calculation and endpoints

The sample size calculation centered on the primary endpoint—the first repositioning success rate, defined as the absence of vertigo and nystagmus after a single attempt of the repositioning maneuver. Assuming a baseline first repositioning success rate of 70% for the traditional Epley maneuver, we anticipated a 20% increase with the modified Epley maneuver. With a significance level of 0.05 and a power of 0.8, the calculated sample size was 28 patients in each group. To account for potential dropouts (estimated at 10%), the sample size was increased to 32 patients per group. Secondary endpoints included the number of repositioning attempts for successful reduction, canal switching rate, repositioning time, and patient tolerance.

### 2.2 Ethical considerations

Approval for the study was obtained from the ethical committee of Wenzhou People’s Hospital (KY-2022-080). The study adhered to the principles of good clinical practice (ICH-GCP), the Declaration of Helsinki, and national laws and regulations regarding clinical studies. Written informed consent was obtained from eligible patients, or in cases of incapacity, approval was sought from a legally acceptable representative (see Table 1).

**Table 1:** Comparison of baseline information of the 2 groups of patients.

Characteristic	Control group	Experimental group	χ2/t/Z	P
Male/Cases (%)	10(31)	9(27)	0.124	0.724
Age/Years	51.59 ± 14.74	7.09 ± 14.3	1.254	0.932
History of vestibular disease/Cases(%)	8(25)	14(42)	2.203	0.138
Disease duration/d	20.69 ± 64.0	6.27 ± 8.0	1.769	0.077
Right posterior semicircular canal/Cases(%)	16(50)	20(61)	0.740	0.390
Left posterior semicircular canal/Cases(%)	16(50)	13(39)	0.740	0.390
Combined Hypertension/Cases(%)	10(31)	8(24)	0.398	0.528
Combine Diabetes/Cases(%)	3(9)	3(9)	0	1
The duration of the latency time/Seconds	0.875 ± 2.091	0.909 ± 2.777	0.056	0.478
Time of nystagmus/Seconds	16.375 ± 8.354	15.485 ± 7.041	0.465	0.322

### 2.3 Subjects

Patients diagnosed with unilateral posterior semicircular canal BPPV at Wenzhou People’s Hospital from January 2022 to October 2022 were included. Inclusion criteria comprised patients aged 20 to 80 years exhibiting vertigo episodes lasting no more than 60 s triggered by a change in head direction relative to gravity. Diagnosis was confirmed through the Dix-Hallpike maneuver, with delayed torsional upbeating nystagmus lasting no more than 60 s. No nystagmus induced by the supine roll test or torsional nystagmus evoked by the supine roll test and cannot be attributed to other diseases (13).

Exclusion criteria encompassed an inability to complete physical therapy due to language comprehension or compliance issues, involvement of horizontal or multiple semicircular canals, and the presence of severe cervical spondylosis, cardiac arrhythmia, heart failure, movement disorders, or upper gastrointestinal bleeding, history suggestive of alternate peripheral or central vestibular disorders including vestibular neuritis, Ménière’s disease, migrainous vertigo, etc., torsional upbeating nystagmus lasting >60 s provoked by the Dix-Hallpike maneuver suggestive of cupulolithiasis (13). A computer-generated randomization sequence divided the 65 eligible patients into the Control and Experimental groups, ensuring no statistically significant baseline imbalances between the two groups (*p* > 0.05) as confirmed by a balance test utilizing standardized mean difference (SMD) (14, 15).

### 2.4 Equipment

**Figure 1:**
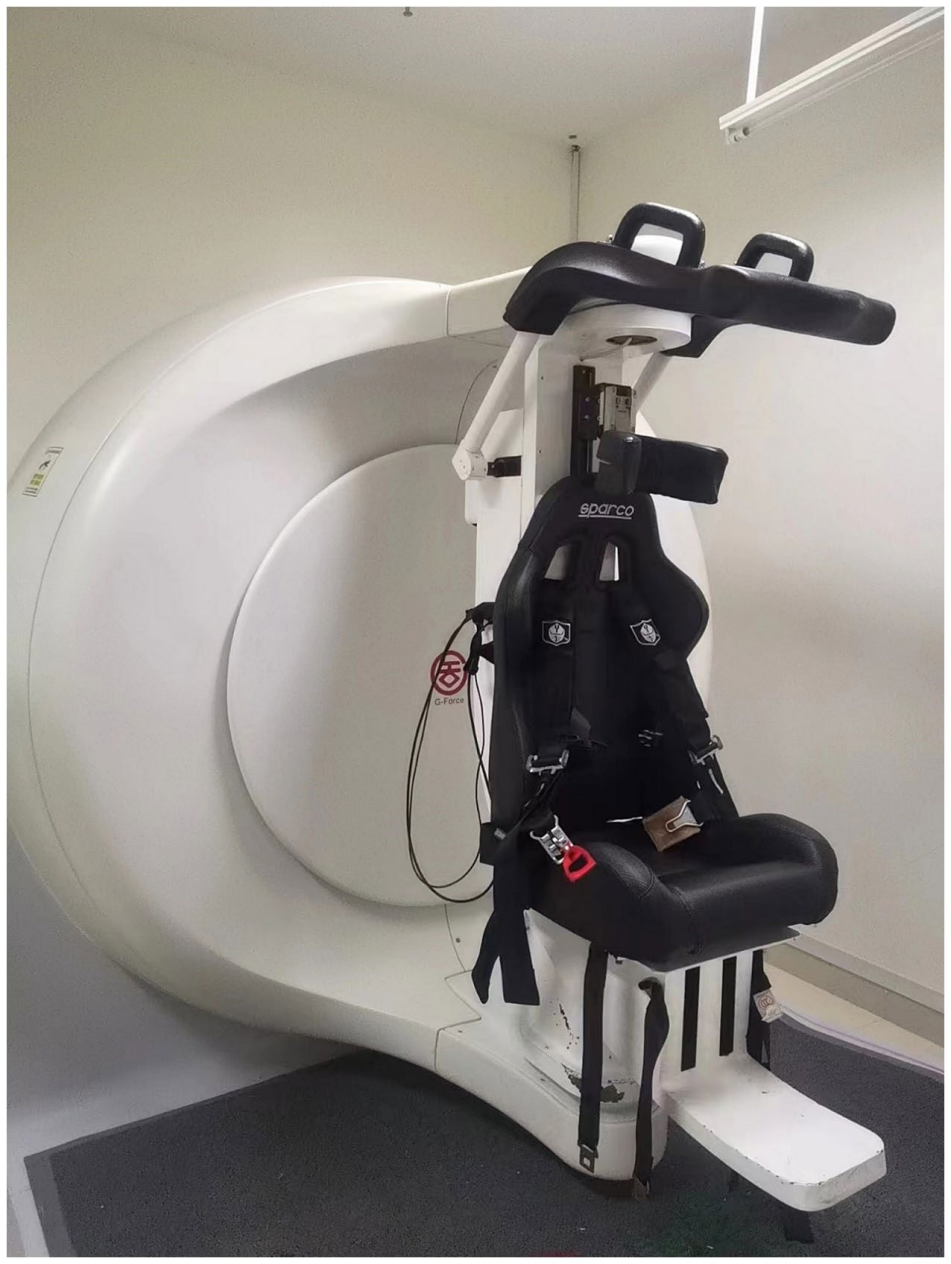
The G-Force swivel chair system.

The G-Force swivel chair system (Figure 1) has a high accuracy and stability for nystagmus detection and recording, with spatial and temporal resolution of 640*480@60 Hz (16). The system we have developed also generates a BPPV virtual simulation model based on the patient’s nystagmus data and repositioning maneuver parameters, which can be used to visually analyze the movement of the otolith in the semicircular canal (17).

### 2.5 Repositioning maneuver

**Figure 2:**
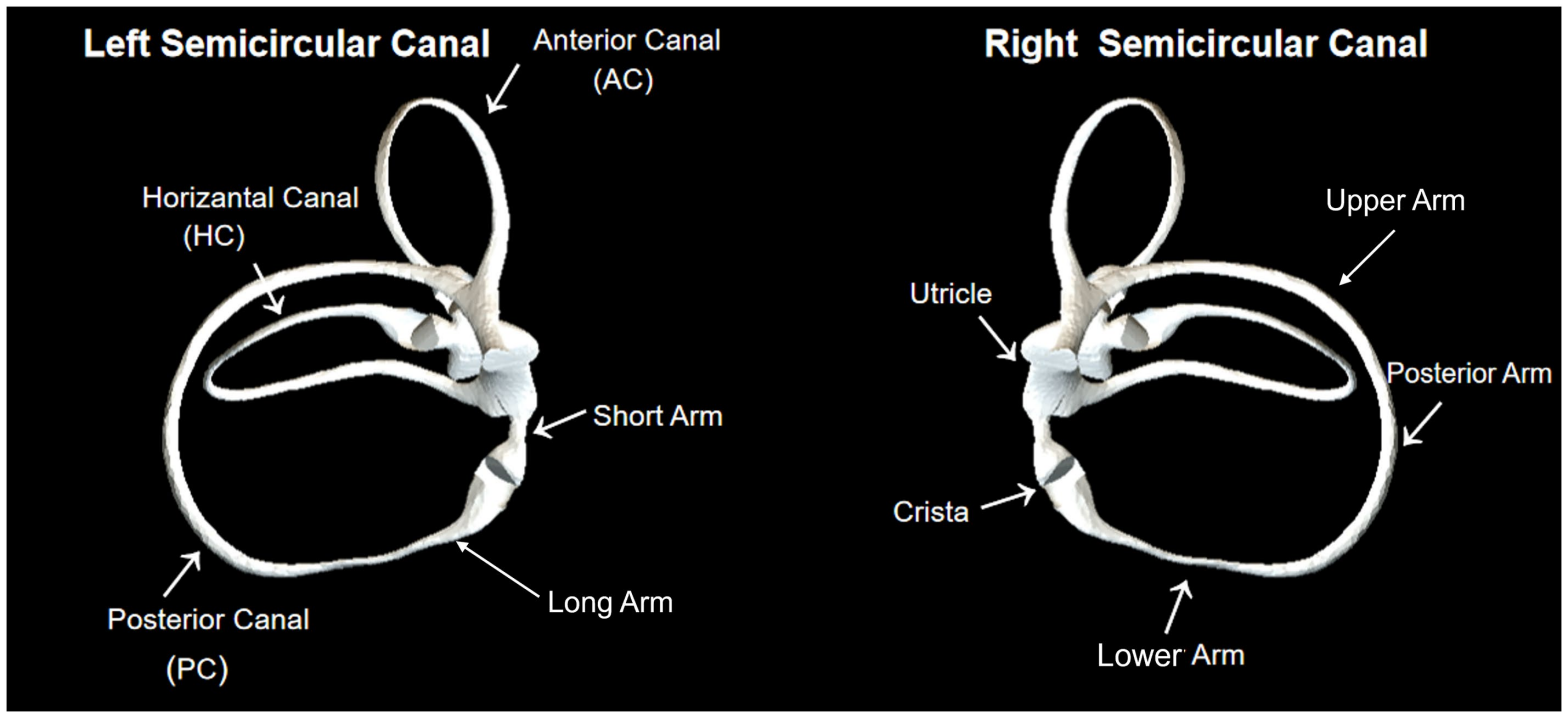
The structure of the semicircular canal shows the anterior, horizontal, and posterior canals. By using the crista as a boundary, the semicircular canal is divided into short and long arms. Besides, the long arm is divided into lower, posterior and upper parts.

**Figure 3:**
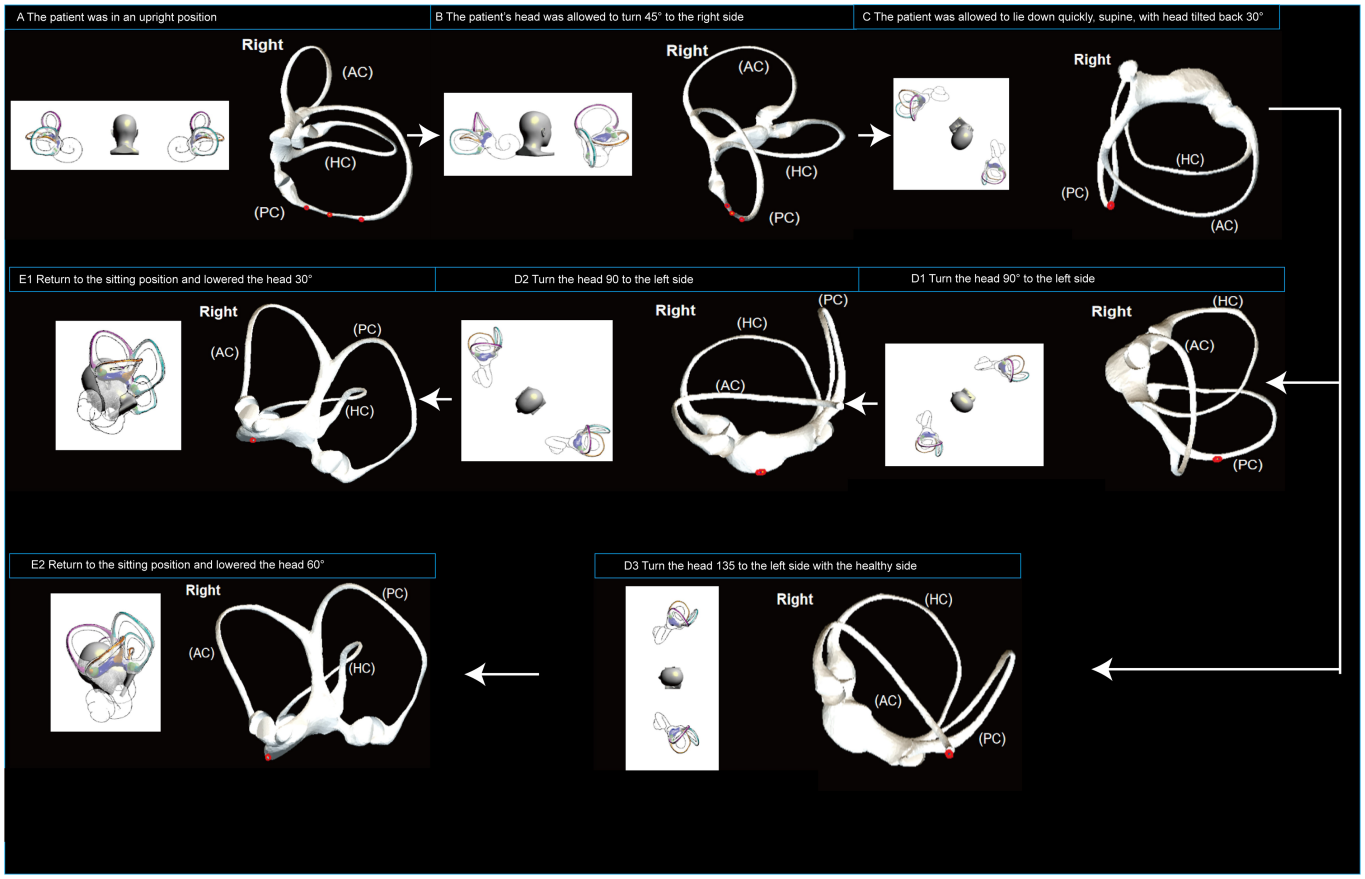
Operational flowchart of the right Epley maneuver and the modified right Epley maneuver. The left side shows the schematic diagram of the head position and the right side shows the corresponding virtual simulation model of the right semicircular canal. Red dots represent otoliths. AC: anterior semicircular canal; HC: horizontal semicircular canal; PC: posterior semicircular canal.

Figure 2 illustrates the structure of the semicircular. Figure 3 illustrates the operational flow of the Epley maneuver and modified Epley maneuver. The Control group underwent the traditional Epley maneuver (Figure 3), while the experimental group received the novel modified Epley maneuver (Figure 3). The operations are as follows:

Right Epley repositioning maneuver. (A) The patient was in an upright position (B) The patient’s head was allowed to turn 45° to the right side (C) The patient was allowed to lie down quickly, supine, with head tilted back 30° and the position was maintained for 1 min. (D1) Turn the head 90° to the left side, keeping the head tilted back and maintained for 1 min. (D2) Turn the head 90 to the left side, keeping the head flat or tilted back, and maintained for 1 min. (E1) Return to the sitting position and lowered the head 30° and held it for 5 min.

Novel modified right Epley repositioning maneuver. (A) The patient was in an upright position. (B) The patient’s head was allowed to turn 45° to the right side. (C) The patient was allowed to lie down quickly, supine, with head tilted back 30° and the position was maintained for 1 min. (D3) Turn the head 135° to the left side with the healthy side lying down and maintained for 5 min. (E2) Return to the sitting position and lowered the head 60° and held it for 5 min.

### 2.6 Observed indicators

The Dix-Hallpike maneuver, performed 5 min after the first repositioning, evaluated the repositioning effect. Patients without vertigo and nystagmus were considered cured. If vertigo and nystagmus persisted or transformed into other BPPV types, the repositioning was deemed ineffective. Each group underwent a maximum of 3 repositioning attempts, with evaluation after 5 min each time. The observed indicators included the success rate of the repositioning maneuvers (1st, 2nd, and 3rd) and the incidence of canal switching.

### 2.7 BPPV virtual simulation model

A BPPV virtual simulation model was employed to visualize and analyze otolith movement during traditional and modified Epley maneuvers (17). Developed using Unity 3D software (version 2020.3) and the NVIDIA physics engine, the model simulated head movements and postural changes based on maneuver parameters (17). Real patient nystagmus data from the G-Force swivel chair system were used for calibration and validation, generating realistic and dynamic images of otolith movement in the semicircular canal under varying head positions (16, 18, 19).

### 2.8 Statistical analysis

Data analysis utilized SPSS 22.0 software, with measurement data expressed as x ± s. The t-test compared the age of the two groups, while the Mann–Whitney U test compared disease duration due to non-normal distribution. χ2 was employed for comparing patient history of vestibular disease, gender, underlying disease, laterality of the involved semicircular canal, and repositioning effect, with a significance level set at α = 0.05.

## 3 Results

### 3.1 Repositioning outcomes

In the Control group, the traditional Epley maneuver successfully repositioned 32 cases. Among these, 20 cases were successfully repositioned on the first attempt, accounting for 63%. Additionally, 6 cases were successfully repositioned on the second attempt (19%), and 2 cases required three attempts for successful repositioning (6%). Unfortunately, 4 cases in the Control group were converted into horizontal semicircular canals, constituting 13% of the cases.

**Figure 4:**
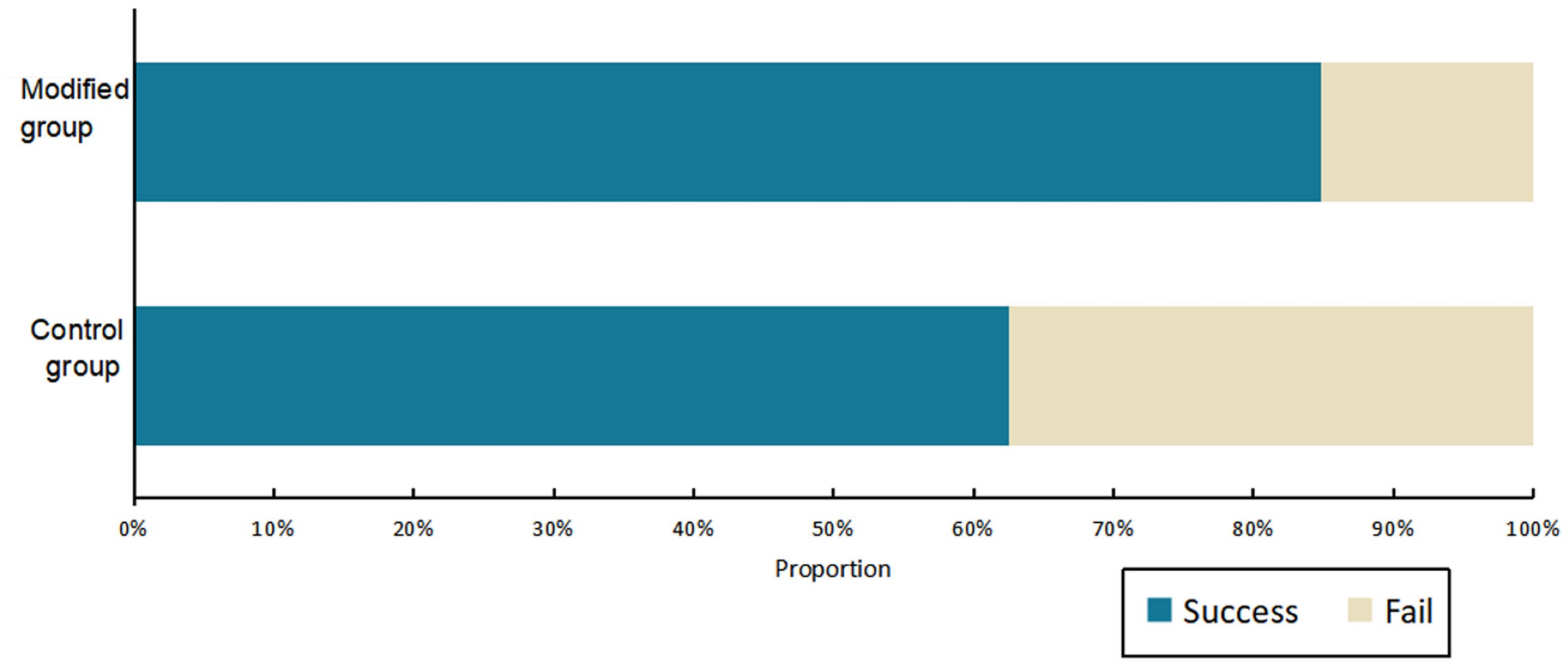
The success rate of first reposition. The success rate was significantly higher in the experimental group than in the Control group. (χ^2^test: *p* < 0.05).

In the experimental group, the modified Epley maneuver successfully repositioned 33 cases. Of these, 28 cases were successfully repositioned on the first attempt, constituting 85%. Moreover, 5 cases were successfully repositioned on the second attempt (15%) (see Table 2). The first repositioning success rate in the experimental group was significantly different from that of the Control group, with the experimental group showing superior performance (χ2 = 4.201, *p* = 0.040) (see Figure 4). Importantly, in the experimental group, resulting in a 100% success rate after two repositioning attempts, while in the Control group, two cases required triple repositioning maneuvers for success. Furthermore, no canal switching occurred in the experimental group. Despite these variations, there was no significant difference in the total repositioning success rate between the two groups (χ2 = 2.498, *p* = 0.114).

**Table 2:** Comparison of the repositioning effect of the 2 groups of patients.

Group	n	Number of first successful repositions/Cases(%)	Number of second successful repositions /Cases(%)	Number of third successful repositions/Cases(%)	Total number of failed repositions/Cases (%)	Total number of successful repositions/Cases (%)
Control group	32	20(63)	6(19)	2(6)	4(13)	28(86)
Experimental group	33	28(85)	5(15)	0(0)	0(0)	33(100)
χ2		4.201	0	0	0	2.498
*p*		0.040	1	1	1	0.114

### 3.2 BPPV virtual simulation model

**Figure 5:**
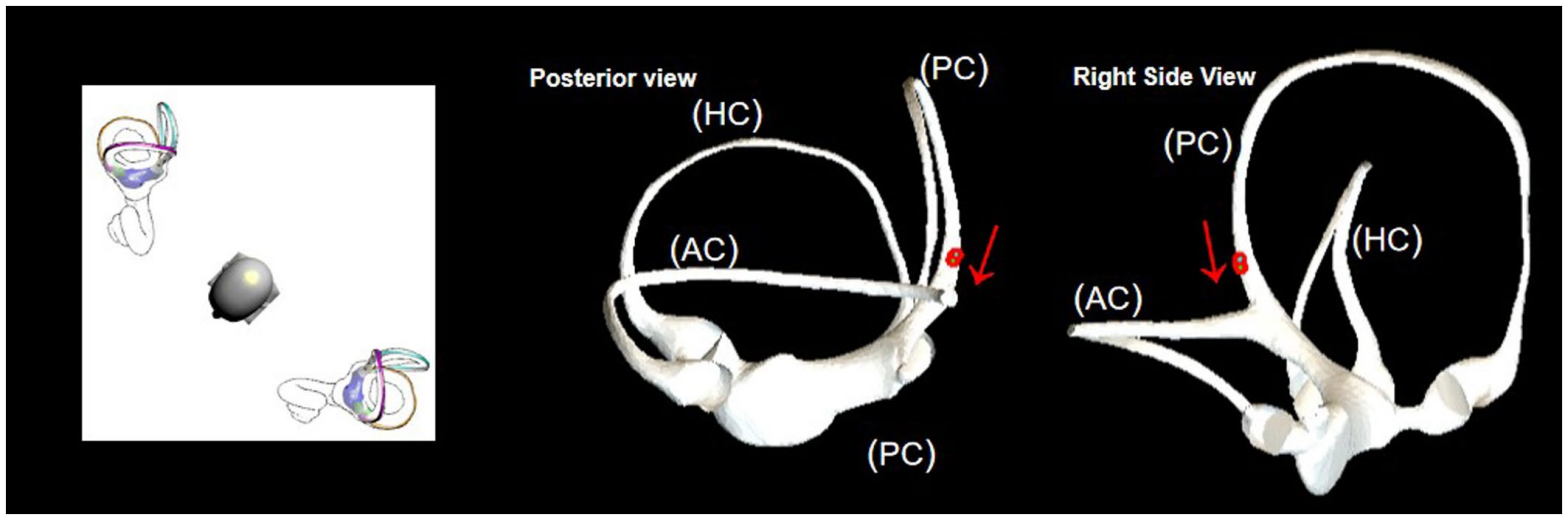
In the head-downward position of the Epley maneuver, the otoliths in the posterior semicircular canal enter the utricle through the common duct. The red arrow represents the direction of otolith advancement.

**Figure 6:**
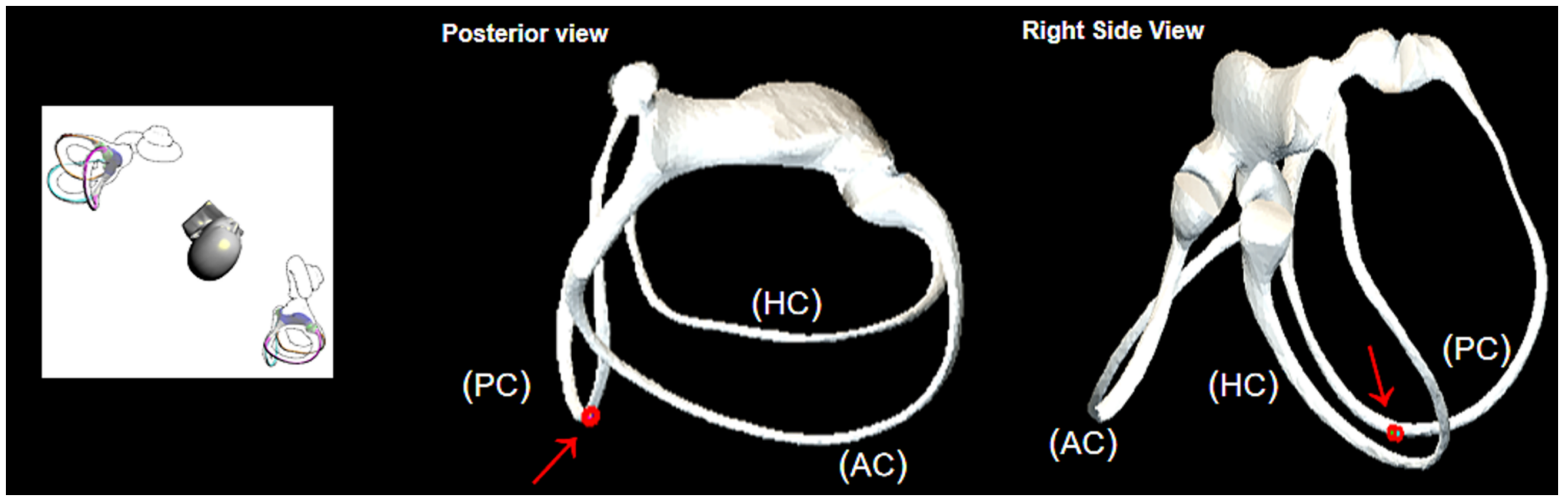
Position of otolith deposition in the supine position. The red arrow indicates where the otolith is obstructed.

The BPPV simulation model illustrated that during the head-down position of the Epley maneuver, the otoliths in the posterior semicircular canal entered the utricle via the common duct (see Figure 5). In the supine position, the otoliths in the posterior semicircular canal were prone to deposition in the posterior arm (see Figure 6).

**Figure 7:**
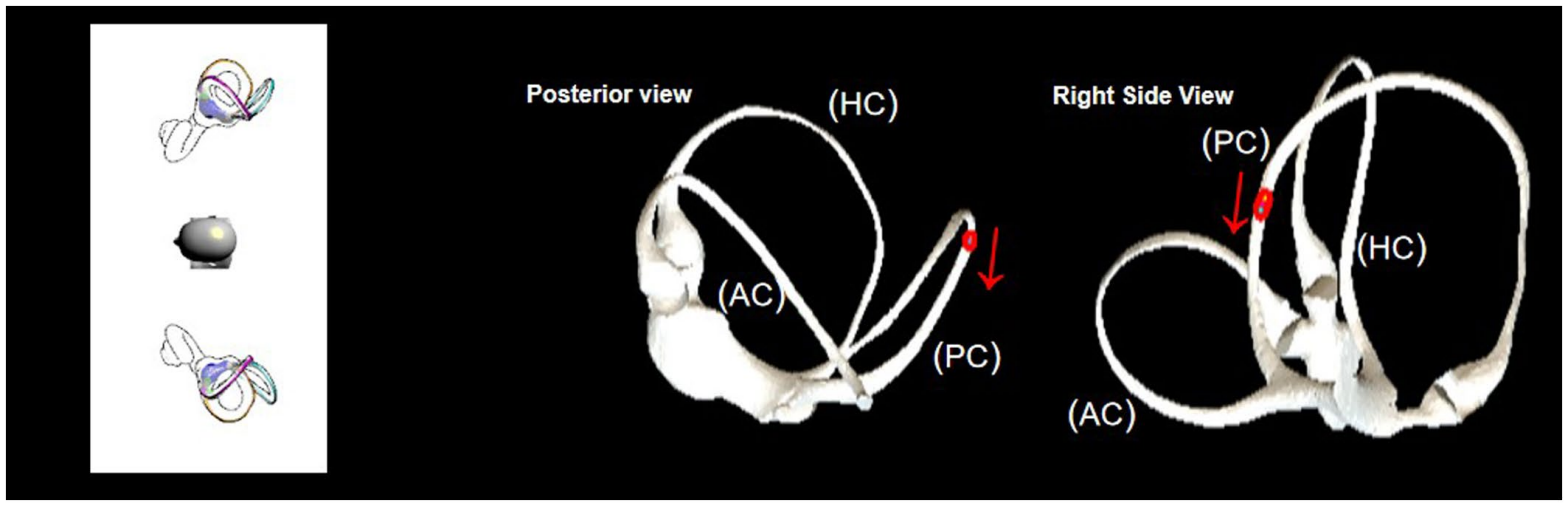
Observation of otolith passage through the obstructed position in the healthy side lying position. The red arrow represents the direction of otolith advancement.

Upon direct transfer of the patient to the healthy side lateral position after the supine position, the simulation model demonstrated that the otolith in the obstructed position moved away from the ampulla. Subsequently, under the influence of gravity, the otolith left the posterior semicircular canal and entered the common crus. This position was found to be more conducive for the otolith to slide into the common duct (see Figure 7). Drawing on clinical experience, extending the retention time in the lateral position of the healthy side to 5 min was deemed sufficient for the otolith to effectively enter the common duct.

## 4 Discussion

The Epley maneuver, a widely utilized repositioning technique for posterior canal benign paroxysmal positional vertigo (PC-BPPV), may encounter challenges such as ineffectiveness or canal switching.

In this study, we introduce a novel modified Epley maneuver and analyze its mechanism using the BPPV virtual simulation model.

This modification involved prolonging the time in the healthy side lying position and the final bowing position. Our findings indicate that the modified Epley maneuver significantly enhanced the single repositioning success rate and reduced the incidence of canal switching in PC-BPPV when compared to the traditional Epley maneuver.

Several factors contribute to the failure or complication of the Epley maneuver for PC-BPPV (10), including anatomical variations, membranous canal stenosis, otolith adhesion, otolith re-entry, incorrect diagnosis, and inadequate repositioning technique (2, 20–23).

1. Anatomical variations of the affected semicircular canal, such as semicircular canal fistula or fracture, which may prevent the complete discharge of otoliths or debris in the expected direction during the head movements (20).
2. Membranous canal stenosis, which may occur when the otoliths or debris partially adhere to the membranous semicircular canal, especially the common crus, causing a narrowing of the lumen and impeding the expulsion of the remaining otoliths or debris (21).
3. Otolith adhesion, which may occur when the otoliths or debris adhere to the cupula or ampulla of the affected semicircular canal, making them resistant to gravity and head movements (22).
4. Otolith re-entry, which may occur when the otoliths or debris that have entered the utricle fall off again and re-enter the semicircular canal, either the same one or a different one, causing recurrent or converted BPPV (23).
5. Incorrect diagnosis, which may occur when the affected side or canal is misidentified, leading to inappropriate repositioning maneuvers or false negative results (2).
6. Inadequate repositioning technique, which may occur when the head movements are not performed with sufficient speed, angle, or duration, or when the postural holding time is too short, leading to incomplete relocation of otoliths or debris (2).

Notably, some patients exhibited no significant movement of otoliths or debris during the head-down position of the Epley maneuver, suggesting an obstruction in the posterior arm of the posterior semicircular canal. This obstruction hindered otolith movement into the utricle, leading to vertigo upon returning to the sitting position. To validate this observation, we utilized a BPPV virtual simulation model, demonstrating that extending the time in the healthy side lying position facilitated otolith movement through the posterior arm, preventing their return to the ampulla and subsequent vertigo.

The model illustrated an obstruction in the posterior arm during the head-down position, impeding otolith passage through the common crus (Figure 5). Transitioning to the healthy side lying position facilitated otolith movement away from the ampulla, aiding their entry into the common crus (Figure 7). Extending the postural holding time in this position enhanced otolith passage through the posterior semicircular canals, particularly past the site of obstruction, preventing their dislodgment.

In a randomized controlled trial involving 65 unilateral PC-BPPV patients, our modified Epley maneuver demonstrated a significant improvement in the single repositioning success rate (85%) compared to the traditional Epley maneuver (63%). Additionally, the canal switching rate was reduced to 0% in the experimental group compared to 13% in the control group, indicating the efficacy and safety of our modification.

Comparisons with other modified Epley maneuvers from existing studies reveal varying success rates. The Semont maneuver achieved success rates of 72–84% and 92–93% after one and two maneuvers, respectively (24, 25). The Modified Epley Maneuver achieved success rates of 76.2–83% and 92–95.2% after one and two maneuvers (25, 26). A shorter variant of Epley’s treatment is the so-called Quick Liberatory Rotation, based on the same principles and technique as Gans maneuver (27), achieved success rates of 81 and 96% after one and two maneuvers, respectively (27, 28).

Our modified Epley maneuver demonstrated a one-maneuver success rate of 85% and a two-maneuver success rate of 100%, suggesting its efficacy in achieving superior treatment outcomes.

In summary, our modified Epley maneuver effectively addresses challenges associated with PC-BPPV by overcoming obstructions in the posterior arm, resulting in more efficient and safer otolith relocation to the utricle. While various modifications of the Epley maneuver have shown improvements, our modification significantly reduces the need for repeated maneuvers, potentially enhancing treatment adherence in BPPV patients.

Despite these promising findings, our study has limitations, including a relatively small sample size that may impact the generalizability of results. Additionally, our study did not encompass patients with bilateral or multiple canal involvement, necessitating further investigation to assess the applicability and efficacy of our modified Epley maneuver for these cases.

## 5 Conclusion

The utilization of the BPPV virtual simulation model emerges as a valuable tool for both studying and refining repositioning maneuvers in benign paroxysmal positional vertigo (BPPV). In particular, the modified Epley maneuver, applicable to patients with posterior semicircular canal BPPV, even those with semicircular canal obstruction, extends the duration of the healthy side lying position. This extension proves beneficial in facilitating the expulsion of otoliths. Our study contributes a novel treatment approach for patients with posterior canal BPPV, particularly those with refractory cases, offering a promising therapeutic option.

## Data availability statement

The original contributions presented in the study are included in the article/supplementary material, further inquiries can be directed to the corresponding author.

## Author contributions

XC: Conceptualization, Formal analysis, Funding acquisition, Investigation, Methodology, Project administration, Resources, Software, Visualization, Writing – original draft. JM: Conceptualization, Formal analysis, Investigation, Methodology, Resources, Software, Writing – original draft. HY: Data curation, Formal analysis, Investigation, Software, Visualization, Writing – original draft. LP: Data curation, Formal analysis, Investigation, Software, Visualization, Writing – original draft. QT: Data curation, Methodology, Resources, Software, Visualization, Writing – original draft. HZ: Formal analysis, Investigation, Methodology, Software, Visualization, Writing – original draft. CW: Supervision, Validation, Writing – review & editing. XY: Conceptualization, Funding acquisition, Project administration, Resources, Software, Supervision, Validation, Visualization, Writing – review & editing.
